# Relaxin-3 Innervation From the Nucleus Incertus to the Parahippocampal Cortex of the Rat

**DOI:** 10.3389/fnana.2021.674649

**Published:** 2021-06-22

**Authors:** Cristina García-Díaz, Isis Gil-Miravet, Hector Albert-Gasco, Aroa Mañas-Ojeda, Francisco Ros-Bernal, Esther Castillo-Gómez, Andrew L. Gundlach, Francisco E. Olucha-Bordonau

**Affiliations:** ^1^Unitat Predepartamental de Medicina, Facultat de Ciències de la Salut, Universitat Jaume I, Castellón de la Plana, Spain; ^2^UK Dementia Research Institute, Department of Clinical Neurosciences, University of Cambridge, Cambridge, United Kingdom; ^3^Centro de Investigación Biomédica en Red de Salud Mental (CIBERSAM), Madrid, Spain; ^4^The Florey Institute of Neuroscience and Mental Health, Florey Department of Neuroscience and Mental Health, The University of Melbourne, Melbourne, VIC, Australia

**Keywords:** amygdala, calcium-binding proteins, hippocampus, neuropeptide, synaptophysin (Syn)

## Abstract

Spatial learning and memory processes depend on anatomical and functional interactions between the hippocampus and the entorhinal cortex. A key neurophysiological component of these processes is hippocampal theta rhythm, which can be driven from subcortical areas including the pontine *nucleus incertus* (NI). The NI contains the largest population of neurons that produce and presumably release the neuropeptide, relaxin-3, which acts via the G_*i/o*_-protein-coupled receptor, relaxin-family peptide 3 receptor (RXFP3). NI activation induces general arousal including hippocampal theta, and inactivation induces impairment of spatial memory acquisition or retrieval. The primary aim of this study was to map the NI/relaxin-3 innervation of the parahippocampal cortex (PHC), including the medial and lateral entorhinal cortex, endopiriform cortex, perirhinal, postrhinal, and ectorhinal cortex, the amygdalohippocampal transition area and posteromedial cortical amygdala. Retrograde tracer injections were placed in different parts of the medial and lateral entorhinal cortex, which produced prominent retrograde labeling in the ipsilateral NI and some labeling in the contralateral NI. Anterograde tracer injections into the NI and immunostaining for relaxin-3 produced fiber labeling in deep layers of all parahippocampal areas and some dispersed fibers in superficial layers. Double-labeling studies revealed that both hippocampal projecting and calcium-binding protein-positive (presumed GABAergic) neurons received a relaxin-3 NI innervation. Some of these fibers also displayed synaptophysin (Syn) immunoreactivity, consistent with the presence of the peptide at synapses; and relaxin-3-positive fibers containing Syn bouton-like staining were frequently observed in contact with hippocampal-projecting or calcium-binding protein-positive neuronal somata and more distal elements. Finally, *in situ* hybridization studies revealed that entorhinal neurons in the superficial layers, and to a lesser extent in deep layers, contain RXFP3 mRNA. Together, our data support functional actions of the NI/relaxin-3-parahippocampal innervation on processes related to memory, spatial navigation and contextual analysis.

## Introduction

Generation and retrieval of explicit memories depends on complex interactions between the neocortex and the hippocampus, which are mediated by the entorhinal cortex (EC) of the parahippocampal region. The EC conveys multimodal sensory information from the neocortex to the hippocampal formation and vice-versa (Burwell, 2000). Processing along these bidirectional projections contributes to memory storage and retrieval inside the medial temporal lobe (Lavenex and Amaral, 2000). A segregation of function occurs in the entorhinal area whereby the medial entorhinal cortex (MEnt) is involved in processing spatial memory through projections coming from the subiculum, and retrosplenial and postrhinal cortices (Hargreaves et al., 2007). In contrast, the lateral entorhinal cortex (LEnt) is involved in odor and object recognition and familiarity, and receives afferents from the perirhinal cortex (PRh) (Wan et al., 1999; Petrulis et al., 2005; Murray et al., 2007; Deshmukh and Knierim, 2011; Cappaert et al., 2014; Witter et al., 2017).

Sensory inputs mainly arising from associative cortex innervate superficial layers (layers II–III) of the EC (Burwell, 2000). Stellate neurons in layer II form the perforant pathway, which synapses on the distal dendrites of granule cells in the dentate gyrus (DG) and CA3 field (Steward and Scoville, 1976). In turn, pyramidal neurons in layer III give rise to the axons of the temporoamonic pathway that synapse on the distal dendrites of the CA1 field and the subiculum (Steward and Scoville, 1976). Conversely, outputs from the CA1 and subiculum fields innervate the deep layers V and VI of the EC, which relay projections back to the superficial layers of the EC (Dolorfo and Amaral, 1998; van Haeften et al., 2003) and to other areas of the neocortex (Cappaert et al., 2014; Witter et al., 2017).

Medial entorhinal cortex and LEnt differentially contribute to specific components of episodic memories. MEnt neurons fire in spatial patterns of grids that tile the environment and display head direction components and boundaries (Hafting et al., 2005; Sargolini et al., 2006). By contrast, LEnt cells do not display tuning in an empty open field, but increase firing in locations previously occupied by discrete objects during previous trials (Hargreaves et al., 2007; Deshmukh and Knierim, 2011).

External capsule function can be modulated by general widespread projections from subcortical afferents including dopaminergic (Loughlin and Fallon, 1984), serotoninergic (Pum et al., 2008) and noradrenergic (Fallon and Moore, 1978) inputs, which modulates excitation/inhibition balance sharpening learning and memory processes. Also neuropeptides, such as neuropeptide S (Shao et al., 2013) and neurotensin (Xiao et al., 2014) have a likely modulatory influence on EC function.

Most of these modulatory systems arise from the brainstem, and a distinct nucleus that provides a general projection to most emotion-related forebrain structures in the rat brain, including the EC, is the *nucleus incertus* (NI) in the pontine tegmentum (Goto et al., 2001; Olucha-Bordonau et al., 2003). The NI contains the largest population of neurons expressing the neuropeptide, relaxin-3 (RLN3) (Burazin et al., 2002; Tanaka et al., 2005; Ma et al., 2007). There are two additional groups expressing RLN3, i.e., a group in the ventral and anterior part of the periaqueductal gray and a cluster of neurons in the substantia nigra pars lateralis (Tanaka et al., 2005; Ma et al., 2007). However, specific RLN3 projections from these groups are restricted to brainstem or thalamic targets (Nasirova et al., 2020). RLN3 is the cognate ligand of the G_*i/o*_-protein-coupled receptor, relaxin-family peptide 3 receptor (RXFP3) (Liu et al., 2003; Chen et al., 2005; Bathgate et al., 2006). The entire temporal cortex receives neuromodulatory GABAergic, cholinergic and glutamatergic afferents from the medial septum and diagonal band complex (MS/DB) (Sotty et al., 2003) which have been linked to the promotion of the hippocampal theta rhythm (Vertes and Kocsis, 1997). In a similar way, the NI and the RLN3/RXFP3 signaling system have been linked to septohippocampal circuits and hippocampal theta rhythm. Electrical stimulation of the NI induced hippocampal theta rhythm and electrolytic lesion of the NI disrupted hippocampal theta induced by stimulation of the *reticularis pontis oralis* (RPO) (Nuñez et al., 2006). Infusion of an RXFP3 agonist into the septal area enhanced theta power in urethane anesthetized rats, and infusion of an RXFP3 antagonist disrupted hippocampal theta induced by stimulation of RPO (Ma et al., 2009a). Moreover, a functional relationship between the firing of RLN3-expressing NI neurons and the phase of hippocampal theta (Ma et al., 2013); and a causal relationship between NI firing and the emergence of hippocampal theta (Martínez-Bellver et al., 2017) have been observed.

Consequently, NI inactivation disrupted spatial learning in the Morris water maze (Nategh et al., 2015) and the passive avoidance test (Nategh et al., 2016); and intracerebroventricular (icv) infusion of an RXFP3 agonist disrupted short-term spatial memory in a T-maze (Albert-Gascó et al., 2017). In addition, RXFP3 deletion in the DG of adult mice resulted in disruption of the continuous spontaneous alternation T-test, suggesting impairment of spatial working memory (Haidar et al., 2017). Furthermore, in rats, NI microstimulation increased locomotor activity (Farooq et al., 2016); and chemogenetic activation of NI neurons increased locomotor activity in the home cage and in a novel environment, in parallel with increased cortical desynchronization, revealing a heightened arousal (Ma et al., 2017).

All these data describe effects of NI projections on the neural systems that control spatial memory, but only the septal (Olucha-Bordonau et al., 2012) and hippocampal (Haidar et al., 2017) RLN3 projections have been functionally explored. The widespread occurrence of anterogradely-labeled NI and RLN3 fibers has been reported (Tanaka et al., 2005; Ma et al., 2007; Smith et al., 2010; Nasirova et al., 2020), but the precise distribution of these fibers in most regions has not been studied in detail. The distribution in the EC is particularly relevant, as different layers and areas of the EC play different roles in the perception of context, generation of spatial cognitive maps, and acquisition and retrieval of episodic memories (Burwell et al., 2004). Our hypothesis is that a particular arrangement of the NI/RLN3 projections within the parahippocampal areas may preferentially target subsets of areas or particular neuronal types in such a way that may subserve specific aspects of the parahippocampal functions. Therefore, we analyzed the distribution of NI/RLN3 fibers in the rat parahippocampal cortex (PHC), relative to maps identified by the presence of calcium-binding protein-containing neurons. We also quantified the occurrence of RLN3 immunoreactivity in synaptophysin (Syn)-immunopositive nerve terminals, as an index of synaptic terminals in the EC. Finally, we examined the occurrence of RXFP3 mRNA in different layers of the PHC.

## Materials and Methods

### Animals

Male Wistar rats (300–400 g, *n* = 49) were used in this study. All protocols were approved by the Animal Ethics Committee of the Universitat Jaume I Castellón (Spain). All procedures were in line with directive 86/609/EEC of the European Community on the protection of animals used for experimental and other scientific purposes and the guidelines on animal welfare issued by the National Health and Medical Research Council of Australia. Details of the experimental protocols employed are provided below (see Table 1).

**TABLE 1:** Summary of the cases (rats) used in the present study.

Treatment	Cases	*N*
CTB tracer injection in LEnt	MS9, MS13, MS15, MS51, MS52, FG37	6
FG tracer injection in LEnt	MS4, MS19, MS25,	3
CTB tracer injection in MEnt	FG36, MS11, MS45, MS46, MS47	5
FG tracer injection in MEnt	FG31, FG28, FG40	3
Giemsa and NADPHd staining	FO1 to FO6	6
BDA injection in NI + double IHC for CBP	T10-12 FH53-54	5
BDA injection in NI + FG injection in the DG	FH10-12	3
RLN3 IHC	NIF2, F70, F77, MIC2	4
FG injection in the DG, CBP + RLN3 IHC	SEC3 to SEC6, FG32, FG34	6
RLN3 and Syn IF and IHC	Naïve 1-3 (N1-3)	3
FG injection in DG + CBP, Syn and RLN3 IHC	SEC10-SEC11	2
RXFP3 mRNA ISH + RLN3 IHC	Ent1-3	3
Total		49

### Tracer Injections

Rats were anesthetized with ketamine (Imalgene 55 mg/kg i.p.; Merial Laboratories SA, Barcelona, Spain) and xylacide (Xilagesic 20 mg/kg i.p.; Lab Calier, Barcelona, Spain) and trephine holes were drilled in the skull. In these studies, we used Fluorogold (FG, 5-hydroxystabilamide; Cat No. 80014, Biotium, Hayward, CA, United States) and Cholera toxin B (CTB, Cat No. 104, List Biological Laboratories Inc., Campbell, CA, United States) as retrograde tracers that were injected through a 1 μl Hamilton syringe attached to a 30-gauge metal injector passing through a 24-gauge guide cannula. Hippocampal and PHC received 200 nl injections of either 2% CTB or 4% FG. The coordinates for injections were (mm) AP −6.8, ML ± 6.8, DV −7.6 for MEnt; AP −6.8, ML ± 5, DV −7.4 for LEnt; AP −6.5, ML ± 6, DV −6.5 for DG, AP −6.5, ML ± 6.8, DV −6.5 for CA3-CA1, relative to bregma and AP −2.6, ML ± 0, DV −7.2 for NI relative to lambda. We also performed anterograde tracer injections of 10% biotinylated dextran amine 70kD (BDA, Molecular Probes, Paisley, United Kingdom) combined with retrograde injections of FG. After the injections, the injectors were left on place for 10 min before removal. The skin was then sutured and a subcutaneous injection of the analgesic, Buprex (0.05 mg/kg, i.p., Lab Esteve, Barcelona, Spain) was administered to prevent postoperative pain. The rats were then left to recover for 7 days before perfusion.

### Brain Fixation and Sectioning

Rats injected with retrograde and anterograde tracers, and naïve rats used for immunohistochemistry (IHC), immunofluorescence (IF) and RNAscope were deeply anesthetized with pentobarbital (Dolethal, 200 mg/kg i.p; Vetoquinol S.A., Madrid, Spain) and transcardially-perfused with saline (250 ml) followed by fixative [4% paraformaldehyde in 0.1 M phosphate buffer (PB), pH 7.4] for 30 min (∼500 ml). Brains were then removed from the skull and immersed in the same fixative for 4 h at 4°C (or 8 h at 4°C for subsequent RNAscope experiments). Brains were cut in the coronal plane at the level of the flocculi using a rat brain methacrylate matrix, to reliably obtain sections from each brain of equivalent orientation. Brains processed for tract-tracing, IHC and IF were immersed in 30% sucrose in 0.01 M phosphate buffered saline (PBS) pH 7.4 for 48 h at 4°C, and coronal sections (40 μm) were collected using a freezing slide-microtome (Leica SM2000R, Leica Microsystems, Heidelberg, Germany). For each brain, 6 series of sections were obtained and collected free-floating in 0.01 M PBS. For RNAscope studies, brains were embedded in 2% agar and 30 μm sections were obtained using a Leica Vibratome VT1200S. In all cases, sections were stored in a cryoprotective solution (30% glycerol, 30% ethylene glycol and 40% 0.1 M PBS, pH7.4).

### Cytoarchitectonics and Chemoarchitectonics

In order to obtain an accurate map of the parahippocampal divisions, Nissl and nicotinamide-adenine dinucleotide phosphate diaphorase (NADPHd) histochemistry was performed. For the Nissl protocol, Giemsa stain was used (Iñiguez et al., 1985). Briefly, six series of sections of different rats were mounted on chrome-alum-coated slides and dried overnight. On the following day, sections were rehydrated and rinsed 2 × 5 min in 0.06 M KH_2_PO_4_ (pH 4.5) at 60°C. Then slides were dipped in a 1/10 solution of Giemsa stock solution (Sigma, Madrid, Spain, cat #GS-500) at 60°C for 12 min. After staining, slides were rinsed in 0.06 M KH_2_PO_4_ at room temperature on a shaker table for 3 × 5 min. After decoloration, slides were briefly rinsed in distilled water and dehydrated in graded ethanol, cleared with xylene and coverslipped with DPX (Sigma-Aldrich).

For NADPHd histochemistry, free-floating sections were rinsed twice in 0.01 M PBS and incubated in a solution containing 0.0125% nitroblue tetrazolium (cat # N5514, Sigma-Aldrich Madrid, Spain) and 0.05% b-NADPH (N7785, Sigma-Aldrich) in 0.01 M PBS for 2 h at 37°C (Vincent and Kimura, 1992). After incubation, the sections were rinsed 2 × 5 min and mounted on chrome-alum-coated slides and air dried overnight. The following day, the sections were rehydrated in distilled water and dehydrated in graded ethanol, cleared with xylene and coverslipped with DPX.

### Chromogenic Double Immunohistochemistry for RLN3 and CB-28kD, PV, CR, or FG

For analysis of RLN3 in nerve fibers in relation to other markers of the parahippocampal cortices, a double-label IHC protocol was used. Briefly, following incubations with primary antisera, initial secondary reactions were completed to obtain a black reaction product delineating RLN3 fibers labeled with a mouse primary antibody, followed by a second series of reactions to obtain a brown reaction product associated with a calcium-binding protein (CBP) or FG. Thus initially, sections were rinsed 3 × 10 min in 0.01 M phosphate-buffered saline (PBS), pH 7.4 and transferred to blocking solution [4% normal donkey serum (NDS), 2% bovine serum albumin (BSA) and 0.2% Triton X100 in 0.01 M PBS] for 1 h at room temperature (RT). Sections were then transferred to incubation media containing 1:10 mouse anti-RLN3 (HK4-144-10); (Kizawa et al., 2003; Tanaka et al., 2005; Watanabe et al., 2011; Ma et al., 2013) and either 1:5,000 rabbit anti-PV (Cat No. PV27, RRID:AB_2631173, Swant, Marly, Switzerland), 1:5,000 rabbit anti-CB-28kD (CB38, RRID:ABAB_10000340, Swant), 1:2,500 rabbit anti-CR (Cat No. 7697, Swant), or 1:3,000 rabbit anti-FG (Cat No. AB153-I, Merck Millipore, Temecula, CA, United States) in PBS containing 2% NDS, 2% BSA and 0.2% Triton X100 overnight. RLN3 and the other neuronal markers were then revealed consecutively (see Table 2). For RLN3, sections were rinsed twice in PBS and incubated in biotinylated secondary antibody (1:200 biotinylated donkey anti-mouse; Cat No. 715-065-150, Jackson ImmunoResearch, West Grove, PA, United States) for 2 h at RT. Sections were then rinsed three times in PBS and transferred to 1:50 ABC (Vectastain, Cat No. PK-6100; Vector Laboratories, Burlingame, CA, United States). After two rounds of rinsing [2 × PBS and 2 × 0.05 M Tris buffer (TB), pH 8.0], the immunolabeling was revealed as a black reaction product by immersing the sections in 0.025% DAB, 0.5% ammonium nickel sulfate, 0.0024% H_2_O_2_ in TB for 20 min. Sections were then rinsed in 0.01 M PBS for at least 2 h. CBP or FG were revealed by incubation in an appropriate biotinylated secondary antibody (1:200 biotinylated donkey anti-rabbit, Cat No. 711-065-152, Jackson ImmunoResearch) for 2 h. Sections were then rinsed twice in PBS and incubated in 1:50 ABC (Vector Laboratories) for 1 h. After rinsing (2 × PBS and 2 × TB, pH 7.6) the immunolabeling was revealed as a brown reaction by incubating the sections in 0.025% DAB, 0.0024% H_2_O_2_ in TB, pH 7.6 for 20 min. Following several rinses in 0.01 M PBS, sections were mounted on chrome-alum gelatin-coated slides, air-dried, dehydrated in graded ethanol, cleared with xylene, and coverslipped with DPX (Sigma-Aldrich, Madrid, Spain).

**TABLE 2:** Percentage of retrogradely CTB labeled neurons after injections in the MEnt (*n* = 4) or LEnt (*n* = 4) that were also positive for RLN3 in each division of the NI. **(B)** Percentage of ipsilateral labeling (from total labeling) in double CTB-RLN3 or single CTB labeled material after tracer injection in the MEnt (*n* = 4) or LEnt (*n* = 4).

(A)				

Injection site	Side	NIc	NId	
MEnt	ipsilateral	63.50	39.97	
	contralateral	53.89	61.61	
LEnt	ipsilateral	57.47	45.00	
	contralateral	51.90	63.06	

**(B)**				

**% Ipsilateral/region**	**MEnt**		**LEnt**	
	**NIc**	**NId**	**NIc**	**NId**

% ipsilateral double-labeling CTB/RLN3	74.87	66.67	68.43	54.61
% single CTB labeling	71.58	68.50	66.89	61.25

### Chromogenic Double Histochemistry for BDA and FG

A consecutive detection protocol was used to visualize anterogradely-labeled fibers and retrogradely-labeled somata. BDA-positive anterograde fibers were processed initially to obtain a black reaction product, and then a regular chromogenic protocol was followed to detect the retrogradely labeled FG-positive neurons. Briefly, sections were rinsed 3 × 10 min in 0.01 M PBS, pH 7.4 and transferred to 1:50 ABC. After two rounds of rinsing (2 × PBS and 2 × TB), BDA was revealed as a black reaction product by immersing the sections in 0.025% DAB, 0.5% ammonium nickel sulfate, 0.0024% H_2_O_2_ in TB for 20 min. Sections were then rinsed for at least 2 h. Next, sections were transferred to blocking solution (4% NDS, 2% BSA and 0.2% Triton X100 in 0.01 M PBS) for 1 h at RT. Sections were then transferred to incubation media containing 1:3,000 rabbit anti-FG in PBS containing 2% NDS, 2% BSA and 0.2% Triton X100, overnight. Sections were then incubated in a biotinylated secondary antibody (1:200 biotinylated donkey anti-rabbit IgG) for 2 h, followed by 2 × PBS rinses, and incubated in 1:50 ABC for 1 h. After further rinsing (2 × PBS and 2 × TB, pH 7.6) FG immunolabeling was revealed as a brown reaction product by incubating the sections in 0.025% DAB, 0.0024% H_2_O_2_ in TB, pH 7.6. Following several rinses in 0.01 M PBS, sections were mounted on chrome alum gelatin-coated slides, air-dried, dehydrated with graded ethanol, cleared with xylene, and coverslipped with DPX.

### Immunofluorescent Detection of Neuronal Markers

For detection of neural marker proteins, sections were rinsed 2 × 10 min and immersed in a blocking media of TBS containing 4% NDS, 2% BSA and 0.1% Triton X-100 for 1 h at RT. Sections were then incubated in primary antibody solution containing 1:10 mouse anti-RLN3 (as above) and either 1:2,500 rabbit anti-PV, 1:2,500 (PV27, RRID:AB_2631173, Swant), 1:5000 rabbit anti-CB28kD (CB38, RRID:AB_10000340, Swant), 1:1,250 rabbit anti-CR, (CR7697, RRID:AB_2619710, Swant), or (1:1000 guinea pig anti- Syn polyclonal, Cat. No. 101004, RRID:AB_1210382, Synaptic Systems, Göttingen, Germany) in TBS containing 2% NDS, 2% BSA and 0.2% Triton X100 for 48 h at 4°C. Sections were then rinsed (3 × TBS) and incubated in 1:200 FITC-labeled donkey antirabbit IgG (Cat No. 711-095-152, Jackson ImmunoResearch) and 1:200 Texas Red-labeled donkey anti-mouse IgG (Cat No. 715-075-150, Jackson ImmunoResearch); for quadruple labeling 1:200 Cy5labeled donkey anti-mouse IgG (Cat No. 715-175-020, Jackson ImmunoResearch) or 1:200 Cy5labeled donkey anti-goat IgG (Cat No. 705-175-003, Jackson ImmunoResearch) in TBS. Sections were then briefly rinsed in 0.01 M PBS and mounted on chrome-alum gelatin-coated slides, air-dried, dehydrated in graded ethanol and coverslipped with DPX.

### Immunofluorescence Combined With *in situ* Hybridization

The distribution of EC *RXFP3* mRNA-positive neurons, in relation to the location of RLN3-positive fibers was assessed using multiplex *in situ* hybridization [RNAscope^TM^; Advanced Cell Diagnostics (ACD); Newark, CA, United States], in combination with IF. After perfusion, brains were postfixed for 18 h at 4°C. Brain sections (30 μm) were collected using a Vibratome (Leica VT 1200S, Wetzlar, Germany) and transferred to a cryoprotectant medium and stored at −20°C.

For the detection of *RXFP3* mRNA, the probes covered ∼1,000 bp of the target mRNA. Sections were mounted on Superfrost Plus Slides (Cat No. 12-550-15, Fisher Thermo Scientific, Hampton, NH, United States) and air dried. The next day, sections were fixed in 4% PFA for 10 min at 4°C, and rinsed in PBS. Once dry, a hydrophobic barrier was drawn around the sections (#310018, ImmEdge hydrophobic PAP pen, Vector Laboratories). Sections were incubated with protease pretreatment-4 (ACD, Cat No. 322340) for 30 min at 40°C. After a distilled water rinse, sections were incubated for 2 h at 40°C with *Rxfp3* mRNA probe (ACD Cat No. 316181). Following incubation, sections were rinsed with wash buffer (ACD Cat No. 310,091) and the signal was amplified with ACD amplifier reagents, according to the manufacturer’s instructions. After several rinses in wash buffer and PBS, sections were incubated with mouse anti-RLN3 1:5 in PBS for 90 min at RT, and rinsed (2 × PBS). Sections were then incubated in 1:200 Alexa488-donkey anti mouse IgG for 90 min at RT, rinsed and coverslipped with FluorSave^TM^ Reagent (Cat No. 345789, Merck-Millipore, Darmstadt, Germany).

### Visualization and Image Treatment

Chromogenic DAB IHC was examined and recorded using an Olympus BX61 microscope equipped with a digital Leica DFC550 camera (Leica Microsystems, Tokyo, Japan). Maps of RLN3, tracer and CBP labeling were constructed using a camera lucida tube attached to a DM750 Leica microscope (Leica Microsystems) at 20× magnification. These maps were scanned and reduced to the final size for reproduction. IF was analyzed with a TCSSP2 laser confocal scan unit, equipped with argon and helio-neon laser beams attached to a DMIRB inverted Leica microscope (Leica Microsystems). For the Cy3 fluorophore, excitation was 433 nm for 560–618 nm emission. For Alexa 488, excitation was 488 nm for 510–570 nm emission. Serial 0.2–1.2 μm sections were captured using Leica Confocal Software (V 2.61). General maps for triple and quadruple labeling were taken at 20× magnification. Details of putative contacts for FG, CBP, Syn, and RLN3 were taken at 40× magnification. All multiple labeling images were acquired sequentially.

For RNAscope analysis, sample 1 μm single scanned sections were tiled for the MEnt and LEnt. Tiled images were magnified to the level of single-cell resolution and a layer was superimposed to indicate single cells expressing *RXFP3* mRNA and fibers containing RLN3. A cell was considered *RXFP3* mRNA-positive when it contained at least 3 positive dots around a DAPI-positive somata (Albert-Gascó et al., 2017, Albert-Gasco et al., 2019), while a nerve fiber was considered RLN3-positive when it displayed 3 puncta (dots) in a row.

## Results

### Cytoarchitectonic Boundaries

In our descriptions of the occurrence and distribution of a RLN3/NI innervation of the parahippocampal gyrus, we have adopted the general boundaries specified in popular atlases of the rat brain (Paxinos and Watson, 2014; Swanson, 2018), which describe the parahippocampal gyrus as occupying the ventral region between the rhinal fissure laterally and a medial indentation medially. In addition, we have considered a transition area just dorsal to the rhinal fissure, the EC, as part of the parahippocampal gyrus. In the present description, we have considered the parahippocampal gyrus as composed of the perirhinal and postrhinal cortices around the rhinal fissure, the two main divisions of the EC, namely, the medial entorhinal (MEnt) and lateral entorhinal (LEnt), and the amygdalohippocampal transition area (AHiTr).

**FIGURE 1:**
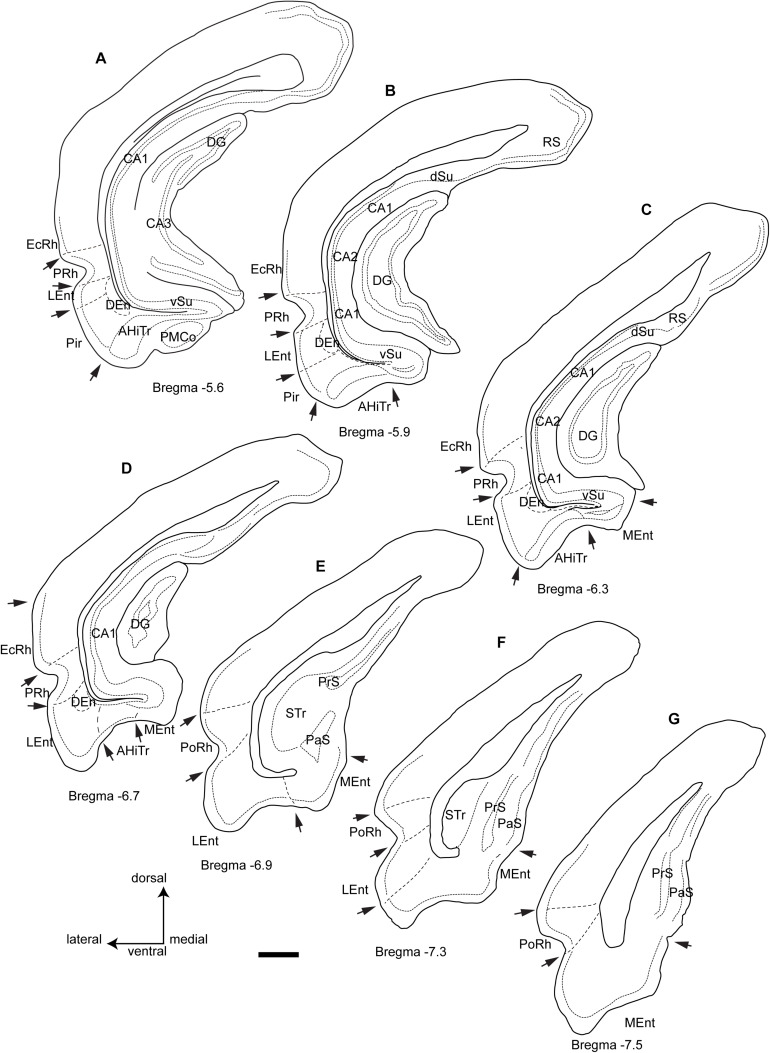
Camera lucida drawings of the rostrocaudal extent of the parahippocampal cortex and the main divisions and landmarks used in this study of the distribution of fibers within cytoarchitectonic defined divisions from rostral level A (Bregma −5.6) to the caudal level G (Bregma −7.5). Arrows point at boundary landmarks between consecutive areas. Calibration bar 1 mm.

In this study, we considered the coronal level −5.6 mm from bregma in the Paxinos and Watson atlas as the most rostral (Figure 1A). At this level the posteromedial cortical amygdala (PMCo) appeared ventrally as an ovoid protrusion in the ventral surface of the cortex. The PMCo was surrounded by the AHiTr, which extended medially to become continuous with the subicular complex. Laterally, the AHiTr finished abruptly at the ventral limit of the LEnt. The LEnt extended along the lateral part of the parahippocampal gyrus, just ventral to the rhinal fissure. In the deepest aspect of the LEnt, the dorsal endopiriform nucleus (DEn) appeared as a cell-poor triangular nucleus.

The second level assessed corresponded to −5.9 mm from bregma (Figure 1B). At this level, the PMCo was no longer present and the AHiTr occupied the ventral aspect of the parahippocampal gyrus. In the medial corner of the parahippocampal gyrus, we identified the rostral tip of the MEnt, which had a cell-poor area lying below the densely packed layer II/III. The dense cell layer of the MEnt was continuous with the subicular area. The Pir cortex was no longer present at this level.

The third level considered corresponded to −6.3 mm from bregma (Figure 1C), where the medial wall of the parahippocampal gyrus was occupied by a wider MEnt and the AHiTr appeared as a shallow notch in the central ventral area of the parahippocampal gyrus. The lateral wall was occupied by the LEnt and a clear border occurred between the AHiTr and the LEnt. The DEn was present in the deepest zones of the LEnt.

The fourth level assessed corresponded to bregma −6.7 mm (Figure 1D), which was the last level containing the DG in the medial aspects of the hippocampus. At this level, the MEnt occupied the medial wall of the parahippocampal gyrus, and in the coronal plane it was composed of vertical and horizontal blades. The central part of the gyrus contained a highly reduced AHiTr, while the LEnt occupied the lateroventral aspects of the parahippocampal gyrus.

The fifth level corresponded to bregma −6.9 mm (Figure 1E), at which the hippocampus was represented by the subicular transition area (STr). In the ventral aspects, subicular-related areas were clearly visible, namely the parasubiculum (PaS), which contained densely-packed small neurons, and the dorsal presubiculum (PrS). The parahippocampal gyrus was divided into a ventromedial aspect for the MEnt and a lateroventral aspect for the LEnt. At this level, the rhinal fissure notch appeared with an open angle, and the cellular organization was different, with a ventral area identified as the ventral postrhinal cortex (vPoRh) and a dorsal area identified as the dorsal postrhinal (dPoRh) cortex.

The sixth level corresponded to bregma −7.3 mm (Figure 1F). In the lateral aspect of the cortex, two areas were clearly identified as PaS, which occupied the superficial area, and a deeper PrS. The MEnt and the LEnt were also clearly differentiated, as the LEnt contained a near single-cell layer II, while the MEnt contained a wider, multineuronal layer II.

The seventh and final level assessed corresponded to bregma −7.6 mm (Figure 1G). At this level, a small indent separated the subicular complex from the parahippocampal gyrus. The STr was absent and the MEnt occupied the ventromedial aspect of the parahippocampal gyrus, characterized by a wide layer II. In the lateral area, the LEnt occupied the ventrolateral aspect and was characterized as a thin layer II. Surrounding the rhinal fissure, the vPsRh and dPoRh occupied the lateral wall of the temporal cortex.

### Neural Tract-Tracer Injections and Retrograde Labeling

**FIGURE 2:**
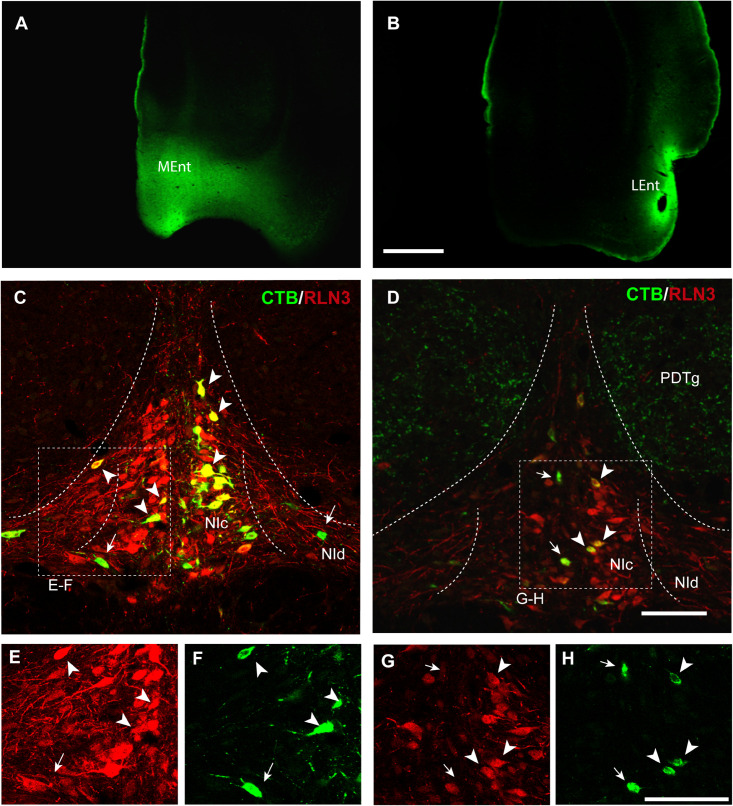
Retrograde labeling of RLN3 neurons in the NI that project to the parahippocampal cortex. This projection is essentially ipsilateral, but some labeling was also observed on the contralateral side. **(A)** CTB injection in the MEnt, case MS9. **(B)** CTB injection in the LEnt, case MS19. Calibration bar 1 mm. **(C)** Retrograde labeling of neurons in the NI after CTB injection in the MEnt with RLN3 IF, case MS9. **(D)** CTB injection into the LEnt resulted in retrograde labeling of NI neurons, most of which displayed RLN3 IF, case MS19. Arrows point to single CTB retrogradely labeled neurons, arrowheads point to double-labeled CTB and RLN3 neurons. **(E,F)** Separated channels for RLN3 and CTB of the square in **(C)**. **(G,H)** Separated channels for RLN3 and CTB of the square in **(D)**. Calibration bar 100 μm.

In the present study, retrograde tracer was injected into the MEnt in six cases – 4 cases received FG and 2 cases received CTB. There were also injections into the LEnt in a total of seven cases – 3 cases received FG and 4 cases received CTB (Table 1 and Figures 2A,B). In all cases, retrograde labeling was observed in both the NI *pars compacta* (NIc) and *pars dissipata* (NId) (Figures 2C,D). We have quantified CTB injections in the MEnt (*n* = 4) and in the LEnt (*n* = 4), where 50–60% of retrogradely-labeled neurons were also positive for RLN3 (Table 2A). Retrograde labeling was concentrated in the ipsilateral side ranging from 60 to 70% of all retrograde labeling and double RLN3-CTB labeling (Table 2B). However, while retrograde labeling was predominantly ipsilateral following injections into the MEnt (Figures 2C,E,F), some retrograde labeling was also seen in the contralateral NI after injections centered in the LEnt (Figures 2D,G,H).

**FIGURE 3:**
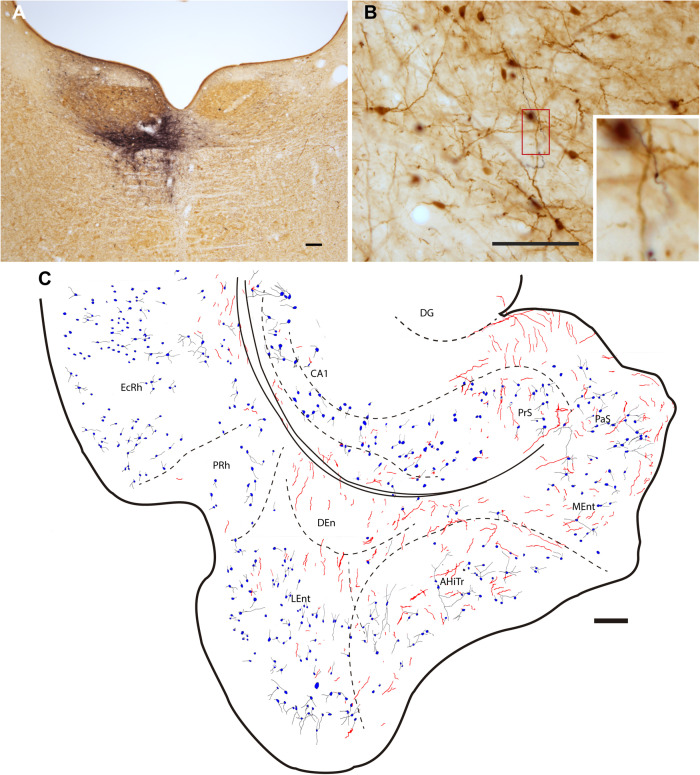
Injection of the anterograde tracer, BDA into one side of the NI and anterograde labeling in the parahippocampal cortex in sections stained for BDA and parvalbumin (PV). **(A)** Injection site in the NI, Calibration bar 200 μm, case F54. **(B)** A section double-stained for BDA and PV illustrating the appearance of anterogradely-labeled fibers (black) and immunoreactive PV neurons in the LEnt and processes (brown). Inset illustrates the boxed area at higher magnification, case F54. Calibration bar 100 μm. **(C)** Camera lucida drawing of the general distribution of anterogradely labeled fibers within the parahippocampal cortex, and the distribution and appearance of PV-labeled somata in case F54. Calibration bar 200 μm.

Injections of the anterograde tracer, BDA, were made into the NI (Figure 3A). Dispersed retrograde labeling was observed in several areas, such as habenula and interpeduncular nucleus (data not shown). No retrograde labeling was observed in the PHC. The resultant anterograde labeling in the PHC was in the form of thin fibers, some displaying typical morphologies of synaptic terminals. In some cases, for example in material labeled for PV immunoreactivity, some of these swellings appeared in close apposition with PV-positive processes (Figure 3B). Anterogradely-labeled fibers were observed throughout the PHC, particularly in deep layers (Figure 3C).

### RLN3 Labeling in Parahippocampal Areas

#### Ectorhinal Cortex

**FIGURE 4:**
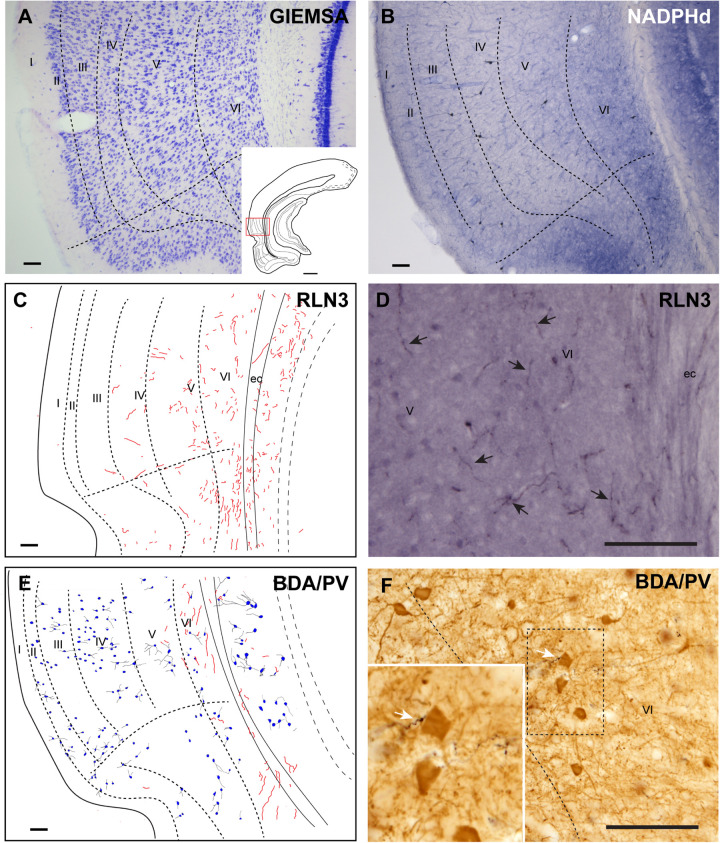
Distribution of anterograde labeling from the NI and RLN3 staining in the ectorhinal cortex. **(A)** Giemsa stain of the boundaries of the ectorhinal cortex. **(B)** NADPHd histochemistry revealed a regular distribution of positive signal, with a slight gradient from deep layers to superficial layers. **(C)** Camera lucida drawings of RLN3-positive fibers in case N1. **(D)** Photomicrograph illustrating the appearance of labeled RLN3 fibers (arrows) in case N1. **(E)** Camera lucida drawing of sections stained for detection of BDA injected into the NI and PV in case FH11. **(F)** Photomicrograph illustrating the appearance of BDA labeled fibers (black) and PV neurons (brown). Arrows indicate close appositions between fibers and neurons in case FH11. Calibration bars **(A–F)** 100 μm.

The ectorhinal cortex (Ect) located just dorsal to the rhinal fissure (Figure 4A) is composed of six regular layers (Figure 4B; Paxinos et al., 2008; Swanson, 2018). NADPHd histochemistry (Figure 4C) revealed that this cortical area is differentiated from the PRh by a weak neuropil reaction, apart from that in layer VI, which displayed more intense NADPHd reactivity. In addition, some dispersed NADPHd-positive neurons of different morphologies were located along layers II to VI. Camera lucida drawings illustrate how RLN3 fibers were preferentially located in layers V and VI (Figure 4D). Anterograde tracer (BDA) injections into the NI produced concentrated ascending fibers in layers V–VI of the Ect, which contained a low density of PV-positive neurons (Figures 4E,F).

#### Perirhinal Cortex

**FIGURE 5:**
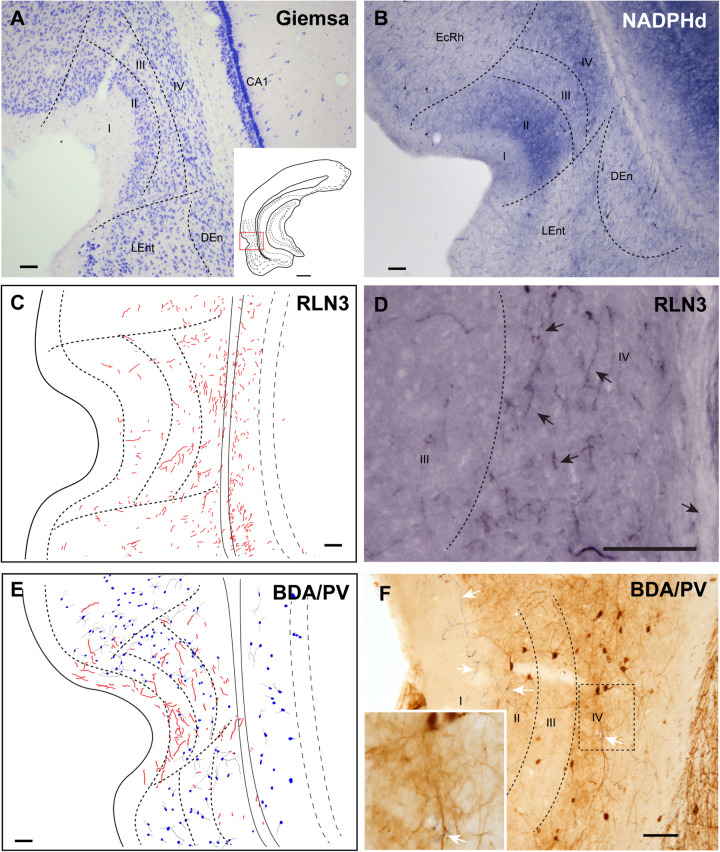
Distribution of anterograde labeling from the NI and RLN3 staining in the perirhinal cortex. **(A)** Giemsa stain of the boundaries of the perirhinal cortex. **(B)** NADPHd histochemistry revealed strong staining in layers II and IV. **(C)** Camera lucida drawings of RLN3-positive fibers in case N1. **(D)** Photomicrograph illustrating the appearance of labeled fibers (arrows) in case N1. **(E)** Camera lucida drawing of sections processed for detection of BDA injected into the NI and PV in case FH54. **(F)** Photomicrograph illustrating the appearance of BDA labeled fibers (black) and PV neurons (brown). Arrows indicate putative contacts between BDA fibers and PV processes in case FH54. Calibration bars **(A–F)** 100 μm.

The cortical area adjacent to the rhinal sulcus was considered as the PRh and contained a wider molecular layer I and three cellular areas of alternative higher and lower cell density (Figure 5A). NADPHd staining was also higher in layers II and IV than in layers I and III (Figure 5B). In line with the presence of RLN3 fibers in deep layers of the Ect, most of the RLN3 fibers were also concentrated in the deeper layers III and IV of the PRh, although some fibers were observed in more superficial layers (Figures 5C,D). Injections of anterograde tracer into the NI resulted in labeling of both superficial and deep layers of the PRh (Figures 5E,F). In this area, there was an apparent mismatch between the distribution of RLN3 (Figure 5D) and anterogradely-labeled (Figure 5E) fibers, as BDA labeling was detected in all layers, whereas RLN3 was concentrated in deep layers. This mismatch may be due to the fact that RLN3 only stains a subset of NI neurons (Lu et al., 2020; Nasirova et al., 2020).

#### Dorsal Endopiriform Nucleus

**FIGURE 6:**
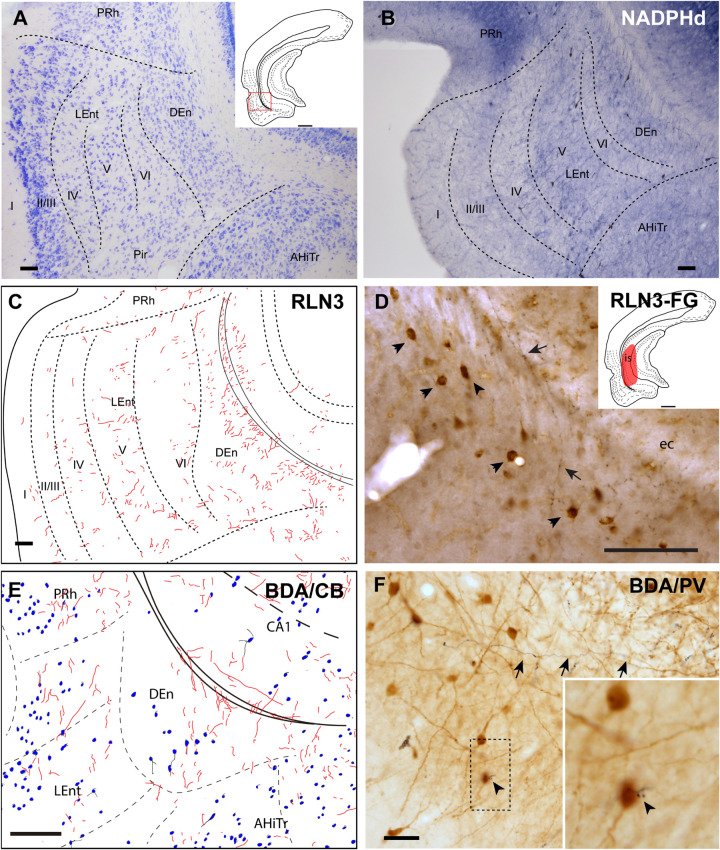
Distribution of anterograde labeling and RLN3 staining in the dorsal endopiriform nucleus. **(A)** Giemsa stain of the boundaries of the DEn. **(B)** NADPHd histochemistry revealed an intense neuropil reaction and some accumulation of positive somata compared with deep layers of LEnt. **(C)** Camera lucida drawings of RLN3 fibers in case N1. **(D)** Photomicrograph illustrating the appearance of RLN3 labeled fibers in the DEn containing retrograde labeling (arrowheads) from FG injection in the CA1 field extending to the DG. Arrow points to a fiber crossing the external capsule (ec) to enter the hippocampus, as part of the perforant pathway in case FG32. **(E)** Camera lucida drawing of sections labeled for BDA injected into the NI and for CB in case FH53. **(F)** Photomicrograph illustrating the appearance of labeling for BDA fibers (black) and PV neurons (brown). Arrowhead points to a close apposition. Arrows point to a long fiber entering the hippocampus via the perforant pathway, case FH54. Calibration bars **(A–F)** 100 μm.

The DEn is located as a ventral extension of the PRh and can be misidentified as the deepest layer of the piriform cortex and LEnt (Figures 1A–E), but it is composed of small oval neurons oriented parallel to the external capsule (ec) (Figure 6A; Paxinos et al., 2008; Swanson, 2018). This nucleus contained a more dense concentration of NADPHd stained fibers, and some dispersed positive neurons (Figure 6B). The DEn contained a high concentration of RLN3-positive fibers (Figure 6C). In rats in which a retrograde tracer was injected into the caudal CA1 hippocampal region, some retrograde labeling was observed in the DEn nucleus, and when double-labeled for RLN3, this area contained RLN3-positive fibers. Some of these fibers could be traced and were observed to cross the EC to enter the hippocampal region, as did the perforant pathway (Figure 6D). In rats in which BDA was injected into the NI, some anterograde labeling was observed in the area containing dispersed CB-positive neurons (Figure 6E). After PV labeling, some apparent contacts were observed between anterogradely-labeled, BDA-positive fibers and PV neurons (Figure 6F).

#### Posterior Extension of the Amygdala

**FIGURE 7:**
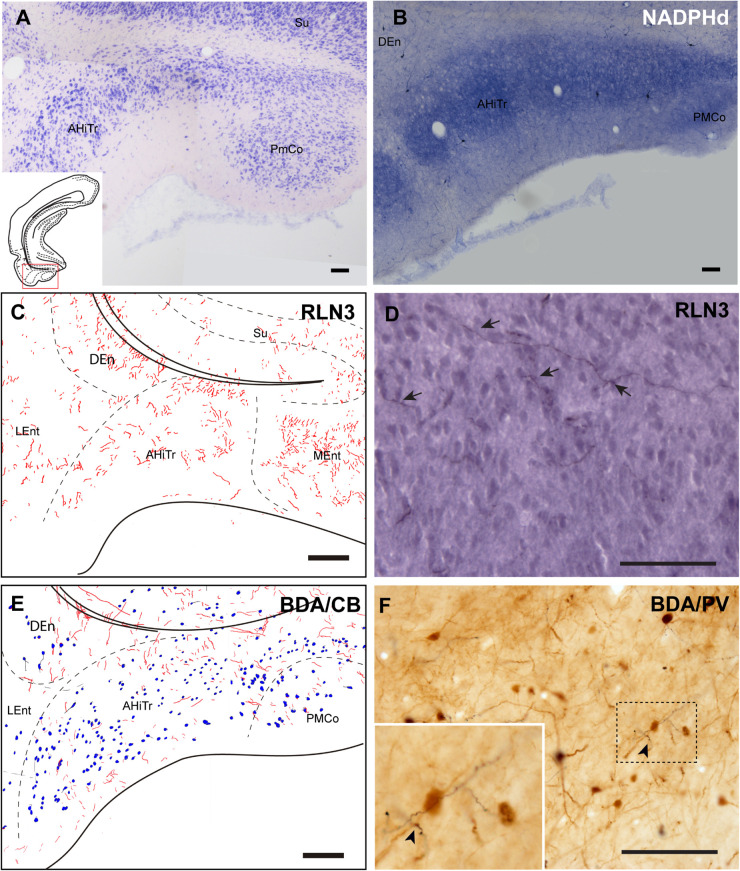
Distribution of anterograde labeling and RLN3 staining in the amygdalohippocampal transition area and posteromedial cortical amygdala. **(A)** Giemsa stain. **(B)** NADPHd histochemistry revealed intense reaction in the amygdalohippocampal transition area and the posteromedial cortical amygdala. **(C)** Camera lucida drawings of RLN3 fibers in case N1. **(D)** Photomicrograph illustrating the appearance of labeled fibers in case N1. **(E)** Camera lucida drawing of sections labeled for BDA injected into the NI and for CB case FH53. **(F)** Photomicrograph illustrating the appearance of labeling for BDA fibers (black) and PV neurons (brown) in deep layers of the AHiTr, case FH53. Arrowhead points at close apposition between fibers and neurons. Calibration bars **(A–F)** 100 μm.

The posteromedial cortical amygdala (PMCo) and the AHiTr are located caudal to the temporal amygdala, enclosed between the MEnt and LEnt and continuous with the DEn (Figures 1A–D). The AHiTr is a curved area containing densely-packed medium to large neurons (Figure 7A; Paxinos et al., 2008; Swanson, 2018). Medially, the posterior levels of the PMCo protrude to the ventral surface of the brain (Figure 7A). NADPHd histochemistry identified an intense labeling of fibers and processes in both the AHiTr and PMCo (Figure 7B).

RLN3 fibers were distributed along the AHiTr, and in some sections were observed to follow the curved morphology of the nucleus and cross the EC, in a similar fashion to the perforant pathway fibers (Figures 7C,D). CB- and PV-positive neurons were dispersed within the AHiTr and PMCo (Figures 7E,F). Injections of BDA into the NI produced dispersed anterogradely-labeled fibers in both nuclei (Figure 7E). Some of these fibers made close contacts with PV-positive processes and somata (Figure 7F).

#### Lateral Entorhinal Cortex

**FIGURE 8:**
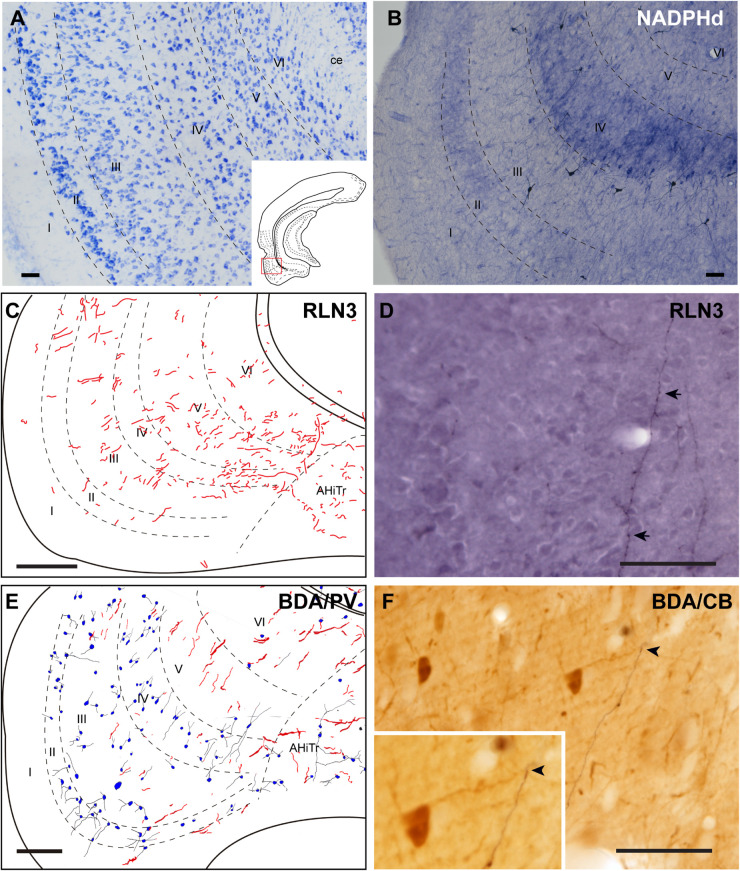
Distribution of anterograde labeling and RLN3 staining in the lateral entorhinal cortex. **(A)** Giemsa stain. **(B)** NADPHd histochemistry revealed alternative layers of dense and sparse reaction. **(C)** Camera lucida drawings of RLN3 fibers. **(D)** Photomicrograph illustrating the appearance of RLN3-labeled fibers (arrows) in layer V, case N1. **(E)** Camera lucida drawing of sections labeled for BDA injected into the NI and for PV, case FH54. **(F)** Photomicrograph illustrating the appearance of labeling for BDA fibers (black) and PV neurons (brown) illustrating a close apposition (arrowheads) in layer V of the LEnt, case FH54. Calibration bars **(A–F)** 100 μm.

The LEnt occupied the anterolateral aspects of the temporal parahippocampal cortices just caudal to the piriform cortex (Figures 1A–F). The LEnt was composed of six cortical layers, and in Giemsa-stained sections, layer II was thin, almost unicellular, and composed of large densely-packed neurons. Layer III contained dispersed, loosely-packed small neurons. Layer IV is also considered as the *lamina dissecans*, a thin, cell-poor layer. Neurons in layer V were large and loosely packed. Finally, layer VI contained small, densely-packed neurons (Figure 8A; Paxinos et al., 2008; Swanson, 2018). In NADPHd stained sections, layers II and V displayed an intense signal (Figure 8B).

There was no concentration of RLN3-positive fibers in any particular LEnt layer, and instead, they appeared in a patchy distribution between dorsal and ventral aspects (Figures 8C,D). In samples stained for PV immunoreactivity after a BDA injection into the NI, labeled cells were concentrated in superficial layers II-IV, avoiding the deep layers V and VI. In these cases, anterogradely-labeled fibers were concentrated in layers V-VI (Figures 8E,F). In samples labeled for CB immunoreactivity and BDA, contacts between anterogradely-labeled fibers and CB neuronal processes were observed (Figure 8F).

#### Medial Entorhinal Cortex

**FIGURE 9:**
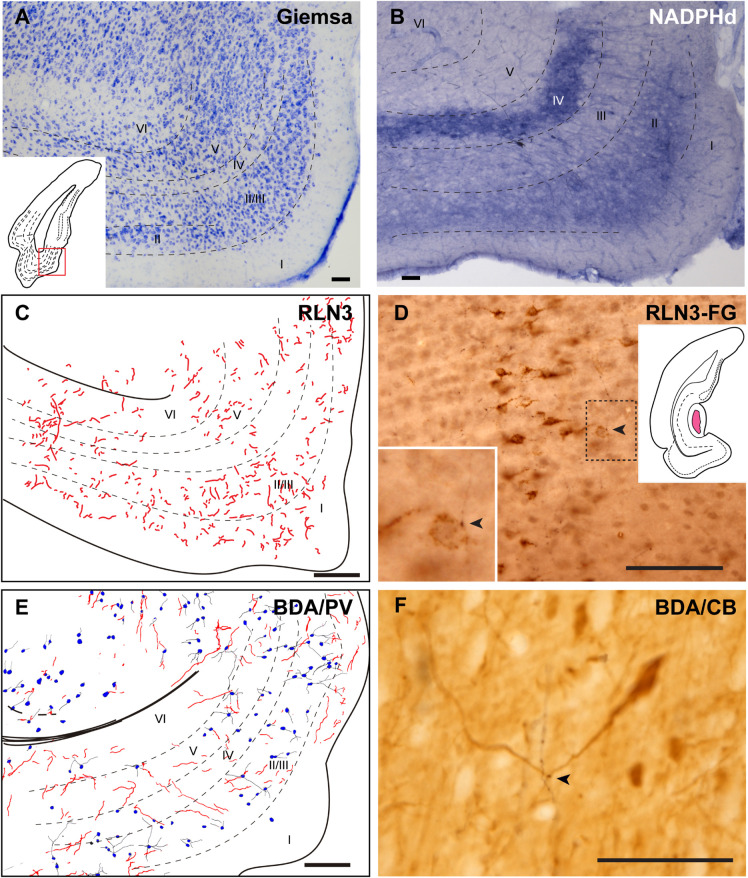
Distribution of anterograde labeling and RLN3 staining in the medial entorhinal cortex. **(A)** Giemsa stain. **(B)** NADPHd histochemistry revealed alternative layers of dense and sparse reaction. **(C)** Camera lucida drawings of RLN3 fibers. **(D)** Photomicrograph illustrating the close apposition between RLN3 fibers and retrogradely-labeled somata from a FG injection into the DG in case FG34. **(E)** Camera lucida drawing of sections labeled for BDA injected into the NI and for PV. **(F)** Photomicrograph illustrating the appearance of labeling for BDA fibers (black) and CB neurons (brown). Arrowheads indicate putative contacts between fibers and neurons.

The MEnt occupied the posteromedial aspects of the temporal parahippocampal cortices and extended from medial to lateral in the region between caudal levels bregma −6.3 to −7.5 mm (Figures 1C–G). The MEnt was composed of six cortical layers. In Giemsa-stained sections, layers II and III merge to form a wider layer. The fusion of these two layers appeared to be in continuity with the parasubiculum. Layer IV was also considered as *lamina dissecans* and was a thin cell-poor layer. Furthermore, layers V and VI appeared merged and contained densely-packed neurons that appeared continuous with the presubiculum (Figure 9A; Paxinos et al., 2008; Swanson, 2018). In contrast, strong NADPHd staining of layer IV was observed. More superficially, although not clear in Giemsa-stained sections, NADPHd staining was stronger in layer II than layer III. By contrast, layers V and VI displayed a similar level of NADPHd staining (Figure 9B). In contrast to the LEnt, in the MEnt RLN3 labeling was stronger in superficial layers, including layer I (Figure 9C). Some of these fibers appeared to be in close apposition to neuronal somata of retrogradely labeled neurons resulting from FG injections into the DG (Figure 9D). Anterogradely-labeled BDA-positive fibers resulting from injections into the NI displayed a similar distribution to RLN3 fibers (Figure 9E). In addition, close appositions between BDA fibers and dendritic processes of PV-positive neurons were observed (Figure 9F).

### Putative Contacts Between RLN3 Fibers and Identified Neurons in Parahippocampal Cortex

**FIGURE 10:**
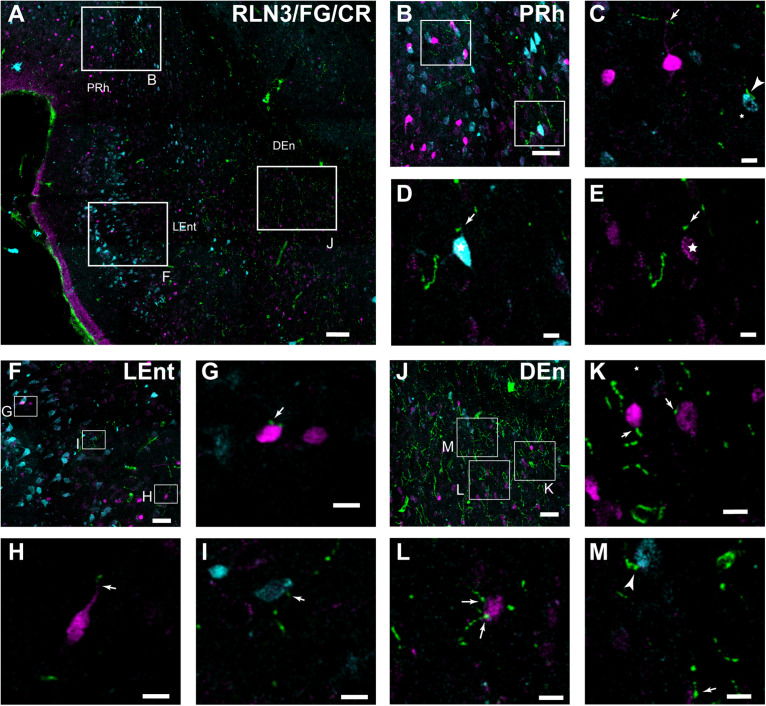
Close contacts in the lateral aspects of the parahippocampal cortex between RLN3 fibers (green channel) and CR-positive neurons (magenta channel) or neurons retrogradely-labeled (cyan channel) by FG injections into the hippocampal CA1 field. **(A)** Overview of an overlay of 10 μm z-stacks in the lateral entorhinal and perirhinal cortices. Calibration bar 100 μm. **(B)** Labeling in a single 1 μm section of the deep layers of the perirhinal cortex. Calibration bar 40 μm. **(C–E)** Higher magnification images of the inset in **(B)**, illustrating RLN3 fibers contacting CR processes (arrow) and retrograde FG labeling (arrowhead) of case FG34, in which the injection site was restricted to the DG. Calibration bars 20 μm. **(F)** Labeling in the superficial layers of the LEnt. Calibration bar 50 μm. **(G–I)** Higher magnification images of the boxed areas in **(F)**, illustrating **(G,H)** contacts with CR neurons and **(I)** FG labeled neurons. Calibration bars 20 μm. **(J)** Labeling in the DEn. Calibration bar 50 μm. **(K–M)** Higher magnification images of the boxed areas in **(J)**, illustrating **(K,L)** contacts with CR neurons and processes and **(M)** FG neurons. Calibration bars 20 μm.

In further studies, the possibility of close appositions between RLN3 fibers and neurochemically identified (CBP-expressing) neuronal types in the PRh, LEnt, and MEnt were examined (Figure 10A; Paxinos et al., 2008; Swanson, 2018). In the deep layers of the PRh, close contacts between RLN3 fibers and CR-positive neurons were observed. In the same material, we observed CR-positive neurons containing FG that had been retrogradely-transported from the DG/CA1 (Figures 10B–E).

In the LEnt, contacts were observed between RLN3 fibers and CR-positive neurons or neurons containing FG retrogradely transported to the area after hippocampal injections (Figures 10F–I). In contrast to the PRh, this area lacked neurons containing FG and CR. Furthermore, in deep (V/VI) layers of the LEnt, and in the DEn, contacts between RLN3-positive fibers and FG- or CR- positive neurons were observed (Figures 10J–M).

### Colocalization of RLN3 and Synaptic Marker Protein in the Entorhinal Cortex

**FIGURE 11:**
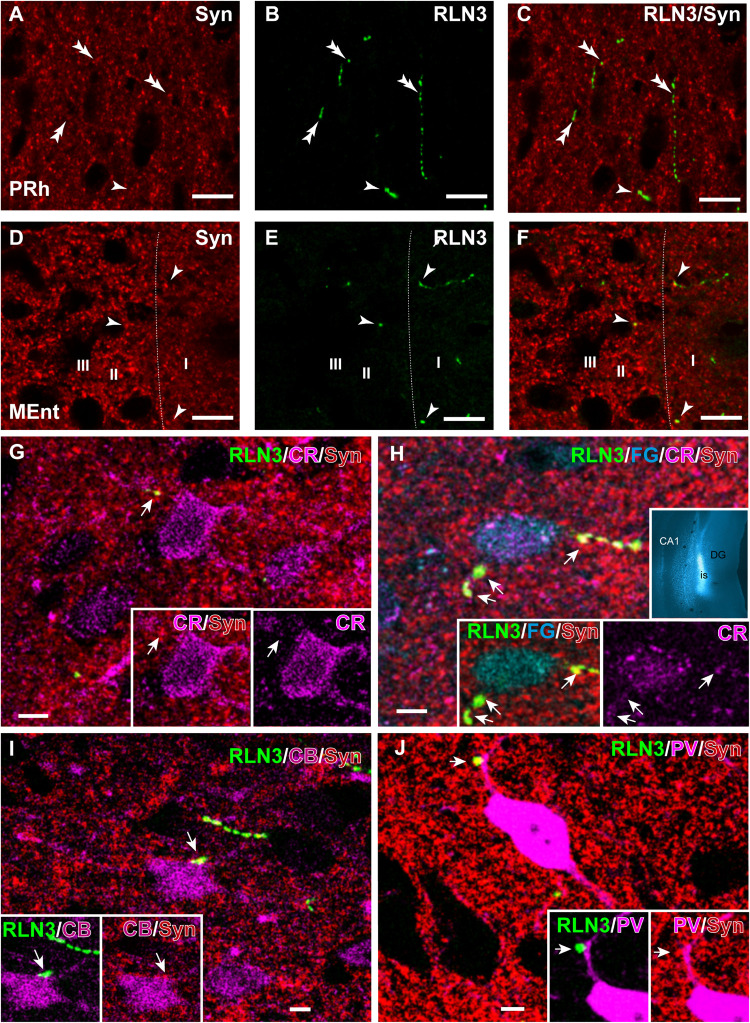
Presence of synaptophysin in RLN3 fibers innervating the parahippocampal cortex. **(A–F)** Confocal images illustrating the presence of synaptophysin (Syn, red) in RLN3 fibers (green). **(A–C)** Sequence of 0.5 μm sections captured with the 40× objective for **(A)** Syn, **(B)** RLN3, and **(C)** merge in the deep layers of the PRh. Calibration bar 5 μm. Colocalization of fibers and putative terminals are indicated by arrowheads and non-overlapping Syn and RLN3 staining is indicated by double arrowheads. **(D–F)** Sequence of 0.5 μm sections captured with the 40× objective for **(A)** Syn, **(B)** RLN3, and **(C)** merged image in the MEnt. Most fibers in this area displayed an overlap of RLN3 and Syn. **(G–J)** Putative close appositions between RLN3 fibers and either CBP-positive (magenta) or FG-positive (cyan) neurons projecting to the dentate gyrus (inset in **H**) of case Sec 6. Syn was observed in the contact. Calibration bars **(G–J)**, 5 μm. **(G)** Confocal single 0.5 μm section of a CR-positive neuron in the MEnt receiving a close apposition from a RLN3 fiber displaying colocalization with Syn puncta. Notably, the Syn and RLN3 puncta also displayed CR IF (arrow). **(H)** Quadruple-labeling for CR, RLN3, FG, and Syn in the superficial layers of the MEnt illustrating a retrograde-labeled (FG injected into the DG, cyan, inset) neuron that is also CR-positive (cyan) and receives RLN3 puncta (green), which are positive for Syn (red). Arrowheads point at puncta that are positive for RLN3, Syn and CR. **(I)** CB-positive neuron receiving close apposition puncta over the soma that is positive for RLN3 and Syn. **(J)** A overlay of 6 μm illustrating a PV-positive neuron in the LEnt receiving a close apposition puncta on a primary dendrite which is positive for both RLN3 and Syn, as shown in the single 0.5 μm images (insets).

In studies aimed at examining the occurrence of an established presynaptic protein, Syn, within RLN3-positive fibers, double labeling studies revealed the colocalization of Syn and RLN3 IF. Syn labeling appeared granular in nature, with puncta 0.5–1 μm in diameter in images acquired at 40× objective magnification and 0.5 μm samples between 2 and 3 μm from the section surface, and the profile of putative neuronal processes and somata were unlabeled (Figures 11A–F). The proportion of RLN3 puncta that were double-labeled for Syn were quantified and ∼60% of RLN3 puncta were also positive for Syn (Table 3). Some CR-positive processes were also positive for RLN3-Syn puncta. Double-labeled RLN3 and Syn puncta were present on cell soma or processes of CR (Figures 11G,H), CB (Figure 11I), or PV (Figure 11J) neurons. Some CR-positive neurons projected to the DG, as reflected by colocalization of CR with retrogradely-transported FG (Figure 11H). Some of the puncta that were positive for RLN3 and Syn were also positive for CR (insets in Figure 11H). It has previously been reported that NI neurons that are positive for RLN3 also are positive for CR (Ma et al., 2007).

**TABLE 3:** Percentage of colocalization of putative RLN3 terminals with Syn-positive puncta in *n* = 3 cases (N1–N3).

% colocalization/region	MEnt	LEnt	PRh	DEn	AHiTr
% double puncta for RLN3 and Syn	62.35	66.66	51.47	66.58	55.99
SEM	5.69	9.87	1.56	4.00	4.43

*Two single optical sections per case for each structure were taken at 40× magnification and between 2–3 microns from the section surface. Then, double-labeled and single-labeled putative RLN3 terminals were counted and averaged for a single case. The standard error of the mean (SEM) was obtained for these three cases.*

### Distribution of RXFP3 mRNA-Positive Neurons and RLN3 Fibers in the Entorhinal Cortex

**FIGURE 12:**
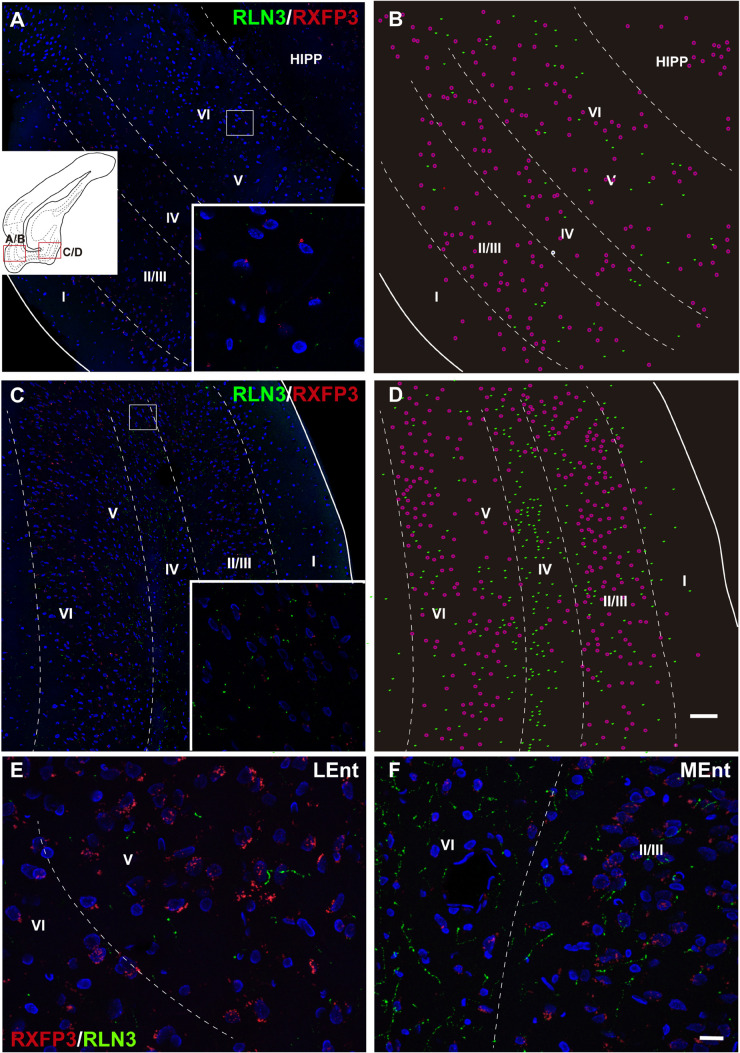
Distribution of *RXFP3* mRNA in neurons of the LEnt and MEnt and their juxtaposition to RLN3 fibers. **(A)** A direct 1 μm single optical section of the LEnt captured with a 20× objective labeled for *RXFP3* mRNA using RNAscope ISH and for RLN3 IF. Inset illustrates the presence of *RXFP3* mRNA (small red dots) surrounding a DAPI-labeled nucleus and several RLN3 fibers (green). **(B)** A plot of the distribution of *RXFP3* mRNA-positive somata, and RLN3 fibers from the section in **(A)**. **(C)** A direct 1 μm single section of the MEnt captured with a 20× objective labeled for *RXFP3* mRNA and for RLN3. Inset illustrates the presence of *RXFP3* mRNA (small red dots) surrounding a DAPI-labeled nucleus and several RLN3 fibers (green). **(D)** A plot of the distribution of labeled *RXFP3* mRNA-positive somata, and RLN3 fibers from the section in **(C)**. Calibration bar **(A–D)**, 100 μm. **(E)** Detail of double-labeling in the inner layers of the LEnt. **(F)** Detail of double-labeling in the inner layers of the MEnt. Calibration bar **(E,F)** 20 μm.

In studies aimed at determining the relative distribution of the RLN3 innervation and RLN3-responsive (RXFP3-expressing) neurons in the EC, we developed a method for the effective co-detection of *RXFP3* mRNA and RLN3 IF, using RNAscope^TM^ *in situ* hybridization and IHC, respectively. In the LEnt, cells expressing *RXFP3* mRNA were distributed evenly along layers II–VI. By contrast, RLN3 fibers were concentrated in layers V and VI (Figures 12A,B). However, *RXFP3* mRNA-positive neurons in the MEnt were denser in layers II/III and VI, with layers IV and V containing a relatively lower concentration of neurons expressing receptor. Conversely, in this same material, it was evident that RLN3-labeled fibers were mainly concentrated in the intermediate layers IV and V, although fibers could be seen in the remaining layers, including layer I (Figures 12C,D). The density of RLN3 fibers was lower in the LEnt (Figure 12E) than in the MEnt (Figure 12F).

## Discussion

This is the first study to describe the anatomical distribution of RLN3 fibers arising from the NI and RLN3 receptor (RXFP3 mRNA) expressing neurons in the PHC of the adult rat. Our analysis revealed a projection from the NI to all areas of the PHC, with a more dense projection to the caudal pole and medial areas. Most anterogradely-labeled fibers resulting from tracer injection into the NI, and the RLN3-positive fibers in the lateral aspects of the PHC were located in the deep layers, whereas in the medial and caudal aspects, RLN3 fibers in the MEnt were observed in more superficial layers. In double-labeled material, most, but not all, RLN3-positive fibers contained the presynaptic protein, Syn, illustrating the likely presence of RLN3-releasing neuronal synapses within the region. While this observation is consistent with the presence of RXFP3 mRNA-expressing neurons in these areas, notably, there is an anatomical mismatch between the precise areas containing a high density of RLN3 fibers and those areas containing the highest density of RXFP3 mRNA-expressing neurons. A possible explanation for this mismatch is the ability of RLN3 released from neurons to travel relatively long distances to activate RXFP3 present on neurons in near adjacent areas, often described as volume transmission, and reported in relation to other peptidergic systems in the amygdala (Pérez de la Mora et al., 2007, 2008; Fuxe et al., 2012).

In this regard, previous studies in rat and mouse brain reported a moderate density of RLN3 fibers in the parahippocampal region, without descriptions of any specific concentration in particular areas (Tanaka et al., 2005; Ma et al., 2007; Smith et al., 2010); while a study of non-human primate brain revealed a high density of RLN3 fibers in the deep layers of the EC (Ma et al., 2009b). In addition, it was observed that RXFP3 mRNA expression and binding sites were observed in several areas of the PHC, including the AHiTr and the DEn in rat and mouse brain (Ma et al., 2007; Smith et al., 2010). Here we report the anatomical distribution of RLN3 and RXFP3, the particular cellular distribution of the receptor in soma, dendrites or axonal terminals has not yet been addressed. In the following sections we discuss the particular effect that RLN3/RXFP3 transmission may have in specific sub-areas of the PHC.

### Perirhinal Cortex

We observed a prominent innervation of deep layers of the PRh throughout its rostrocaudal extent. Considerable data obtained in rodents and non-human primates has led to the view that the PRh encodes experience-dependent features of objects acquired in a particular behavioral context (Miyashita, 2019). In addition, connective and functional data have revealed that this area can be divided into a rostral part, the PRh proper and a caudal part, the postrhinal cortex (PoR). However, we were not able to discriminate between the distributions of NI/RLN3 fibers in these two regions. The PRh proper preferentially projects to the LEnt, while PoR projects to both LEnt and MEnt (Burwell and Amaral, 1998). The PRh also receives afferents from polymodal association areas, while the PoR receives projections from visuospatial processing areas (Furtak et al., 2007). Finally, while the PRh proper is mainly interconnected with the amygdala, the PoR is preferentially interconnected with the dorsal thalamic nuclei (Furtak et al., 2007). Single-unit recordings from the PoR reflect an egocentric relationship of the animal to the geometric center of the space and at the same time an allocentric configuration of the head direction cells related to contextual references (LaChance et al., 2019). Thus, the activation of the NI may enhance the capacity for acquiring these features during active exploration. Recent experiments in mice involving optogenetic stimulation of NI GABAergic neurons during context conditioning revealed that NI neuron activity disrupted context conditioning, while inhibition of these cells during acquisition resulted in context conditioning enhancement (Szőnyi et al., 2019). These observations have been associated with an inhibitory effect of NI neurons on somatostatin interneurons in the CA1 field of hippocampus (Szőnyi et al., 2019), but the perception of the context may be fully or partly initiated in the PRh pathway via the entorhinal-hippocampal perforant pathway (Liu and Bilkey, 1996, 1997).

### Dorsal Endopiriform Nucleus

The DEn has been considered as the ventral extension of the claustrum and part of the claustroamygdalar complex (Medina et al., 2004; Binks et al., 2019; Smith et al., 2019). This chemoarchitectonic differentiation of the claustrum and DEn is associated with a differential connectivity whereby the dorsal claustrum is reciprocally connected with the neocortex, while the DEn is connected to allocortical areas, including regions of the hippocampus and amygdala (Dinopoulos et al., 1992; Behan and Haberly, 1999; Watson et al., 2017). In the current study, NI and RLN3 fibers in the DEn appeared continuous with dorsal claustral fibers observed by anterograde labeling (Olucha-Bordonau et al., 2003) and staining of RLN3 fibers in rat (Tanaka et al., 2005; Ma et al., 2007), mouse (Smith et al., 2010) and non-human primate (Ma et al., 2009b). The DEn receives dense projections from neurons in several areas of the brainstem that express neuropeptide-S as a neurotransmitter (Meis et al., 2008); and parallels can be drawn between the RLN3 and neuropeptide-S systems, as excitatory, neuropeptide-S projections to the DEn have been implicated in anxiety and fear processes triggered by context, but not by a cue (Meis et al., 2008). A high concentration of kappa opioid receptor has been also observed in the claustro-endopiriform complex (Slowe et al., 1999; Chen et al., 2020). Although there is no direct evidence of the involvement of opioid transmission within the DEn in a particular behavior, hallucinogenic and hyperactivity effects of several kappa agonists have been described (Chavkin et al., 2004; Ansonoff et al., 2006; Chen et al., 2020). Thus, parallel ascending projections to DEn may adapt its function to the particular situation that triggers activation of a particular peptidergic system.

### Amygdalohippocampal Transition Area

The deep layers of the AHiTr area are targeted by fibers arising from the NI. These fibers contact retrogradely-labeled neurons in the area that project to the hippocampus. The AHiTr is continuous with ventral hippocampus structures, in an order of CA1, subiculum and AHiTr area (Canteras et al., 1992). Neurons in this continuum express high levels of phosphodiesterase 11 (PDE11) (Kelly, 2017). Social isolation decreased PDE11, which, in turn, impaired social memory formation (Hegde et al., 2016). The primary action of RXFP3 activation is to reduce cellular cAMP levels (Liu et al., 2003), which is a similar effect to the enzymatic activity of PDE. In contrast, there is a prominent projection arising from the AHiTr that targets the medial preoptic area and is related to agonistic behavior of adult mice toward pups (Sato et al., 2020). Thus, the projection from the NI to the AHiTr could be related to social behavior. In fact, previous experiments from our laboratory revealed that icv injection of an RXFP3 agonist resulted in specific disruption of social recognition (Albert-Gascó et al., 2018); and an excitotoxic lesion of the NI resulted in disruption of the social recognition test in the 3-chamber maze (García-Díaz et al., 2019).

### Entorhinal Cortex

In this study, we observed a concentration of RLN3 fibers in deep layers of the LEnt and in superficial layers of the MEnt. By contrast, RXFP3 mRNA-positive somata were more concentrated in the outer layers of both MEnt and LEnt. Indeed, there is a general mismatch between the location of RLN3 fibers and RXFP3-expressing neurons in rat brain. In many cases, the fibers are not in close proximity to the latter cells. For example, in the rat amygdala, RLN3 fibers are concentrated in the medial amygdala (Santos et al., 2016) where RXFP3 mRNA is also expressed (Albert-Gascó et al., 2018), but the highest concentration of RXFP3 is located in the neighboring central nucleus, which lacks RLN3 fibers (Ma et al., 2007; Albert-Gascó et al., 2018). This differential location of fibers and receptors may subserve fast and slow actions of the peptide ligand. This is the case for the action of cholecystokinin (CCK) in the amygdala. CCK fibers are concentrated in the central amygdala, but not in the basolateral amygdala, where a high concentration of CCK2 receptors are present (Pérez de la Mora et al., 2007). In the absence of danger, continuous delivery of CCK into the central amygdala can reach the receptors in the basolateral amygdala and maintain a certain level of arousal (Pérez de la Mora et al., 2007). As yet, no such tonic effect has been investigated for RLN3 in the amygdala or the hippocampus.

In general, it is assumed that neurons in superficial Ent layers relay information from the associative cortex to the hippocampus, while deep layers convey projections from the hippocampus back to the cortex (Witter et al., 1989, 2017; Cappaert et al., 2014). Thus, evidence for the highest concentration of NI/RLN3 fibers in the superficial layers of the MEnt suggests a maximal impact of a direct effect of the NI on the pathway innervating the hippocampus, to influence cognitive map formation. In this respect, it is important to note the ability of NI neurons to drive hippocampal theta rhythm (Nuñez et al., 2006; Ma et al., 2009a, 2013; Martínez-Bellver et al., 2017). During the exploration of a new environment hippocampal neurons synchronize at theta frequency. Under these conditions, a cognitive map of place cells is formed and a relevant feature of place cells is theta precession, which involves advancing the firing at a previous phase of the theta cycle, as the animal is passing through the place field (O’Keefe and Recce, 1993). At this stage, whether the NI and the associated RLN3/RXFP3 system participate in the generation and/or modulation of cognitive maps has not been analyzed. New tools based on vectors containing specific promotors either for RLN3 or RXFP3 may help decipher the specific contribution of the RLN3/RXFP3 signaling system in each sub-area of the PHC.

### Functional Considerations

A relevant aspect to be considered is the situation(s) in which the NI projection to the PHC may modulate its function. The rat NI is characterized by a high density of CRF_1_ receptors (Bittencourt and Sawchenko, 2000; Van Pett et al., 2000); and exogenous CRF specifically activates RLN3-positive neurons in the NI (Ma et al., 2013). NI neurons also express a range of other transmitter and peptide receptors, including dopamine receptors (Kumar et al., 2015), and orexin and MCH receptors (Blasiak et al., 2015; Kastman et al., 2016), consistent with a key role in adaptation of parahippocampal function to a particular condition of stress and arousal.

Notably, the RLN3 innervation of the parahippocampal area targets the superficial layers of the MEnt, which is centrally involved in the construction of neural cognitive maps. Manipulations of the RLN3/RXFP3 system in the medial septum and hippocampus result in alterations of spatial working memory and navigation (Ma et al., 2009a; Albert-Gascó et al., 2017; Haidar et al., 2017, 2019), but the precise role of the RLN3 innervation of the EC in these tasks has not been determined. In light of evidence that the NI/RLN3/RXFP3 system is directly involved in arousal mechanisms via the integration of CRF and orexin signals, the proposed circuit may regulate learning processes in specific arousal contexts.

## Conclusion

The NI projections to the PHC constitute a topographically organized innervation that preferentially targets deep areas of a continuum that runs laterally from the PRh to the subicular area of the hippocampus. The NI pathway, by targeting all of these areas, may produce modulatory effects on the entire PHC, and influence a wide variety of behaviors. For example, when a rat explores an object, the NI projections to the LEnt cortex may be activated, while during an interaction of an adult rat with a pup, the NI projections to the AHiTr may be activated. Finally, NI projections to the MEnt cortex may modulate the processes for generating or retrieving grid cell patterns. These possibilities warrant further investigation in future experimental studies.

## Data Availability Statement

The raw data supporting the conclusions of this article will be made available by the authors, without undue reservation, to any qualified researcher.

## Ethics Statement

This animal study was reviewed and approved by the Animal Ethics Committee of the Universitat Jaume I of Castellón.

## Author Contributions

FO-B: conceptualization. CG-D, IG-M, and FO-B: experimental procedures – tracing and mapping. CG-D, IG-M, EC-G, FR-B, and AM-O: experimental procedures – confocal analysis. CG-D, FR-B, HA-G, AG, and FO-B: experimental procedures – RNAscope design, labeling, and analysis. CG-D, IG-M, HA-G, and FO-B: writing – original draft preparation. HA-G, EC-G, AG, and FO-B: writing – review and editing. CG-D, IG-M, AM-O, FR-B, EC-G, AG, and FO-B: figures and tables – preparation and editing. All authors approved the final manuscript.

## Conflict of Interest

The authors declare that the research was conducted in the absence of any commercial or financial relationships that could be construed as a potential conflict of interest.

## Abbreviations

AHiTr: amygdalohippocampal transition area
alv: alveus
BDA: biotinylated dextran amine 70kD
CA1-3: cornu ammonis fields 1–3
CB: calbindin-28kD
CBP: calcium-binding protein
CR: calretinin
CTB: cholera toxin B subunit
DAB: diaminobenzidine
DEn: dorsal endopiriform nucleus
DG: dentate gyrus
EC: entorhinal cortex
ec: external capsule
Ect: ectorhinal cortex
FG: fluorogold
GABA: γ-aminobutyric acid
HPC: hippocampus
IHC: immunohistochemistry
IF: immunofluorescence
LEnt: lateral entorhinal cortex
MEnt: medial entorhinal cortex
NADPHd: nicotinamide adenosine dinucleotide phosphate diaphorase
NI: nucleus incertus
NIc: nucleus incertus pars compacta
NId: nucleus incertus pars dissipata
PaS: parasubiculum
PBS: phosphate buffered saline
PDTg: posterodorsal tegmental nucleus
PHC: parahippocampal cortex
PMCo: posteromedial cortical amygdaloid nucleus
PoR: postrhinal cortex
PRh: perirhinal cortex
PrS: presubiculum
PV: parvalbumin
RLN3: relaxin-3
RS: retrosplenial cortex
RXFP3: relaxin-family peptide 3 receptor
Syn: synaptophysin
TB: Tris buffer.
